# Supplementary material for: Out-of-School Sports Participation Is Positively Associated with Physical Literacy, but What about Physical Education? A Cross-Sectional Gender-Stratified Analysis during the COVID-19 Pandemic among High-School Adolescents
Source: Children (Basel). 2022 May 20;9(5):753. doi: 10.3390/children9050753 (PMC9139184; doi:10.3390/children9050753)
Supplement: Supplementary file 1 [file children-09-00753-s001.zip › children-1712893-supplementary.pdf]

children

Table S1. Descriptive statistics and differences between groups based on sport participation—total sample.

	Athletic Boys (n = 69)		Non Athletic Boys (n = 38)		t-Test/MW Test	
	Mean	Std.Dev.	Mean	Std.Dev.	t-Value/Z Value	p
Body height (cm)	174.59	9.94	171.40	8.70	2.96	0.001
Body mass (kg)	67.21	14.23	62.59	12.73	2.96	0.001
Body mass index	21.90	3.37	21.21	3.41	1.75	0.08
Broad jump (cm)	196.79	32.96	171.46	29.24	7.04	0.001
Sit and reach (cm)	10.51	8.11	11.71	15.90	−0.80	0.42
Sit-ups (repetitions)	61.89	12.24	51.50	11.07	7.70	0.001
Beep test (level)	10.82	3.00	8.28	2.42	8.13	0.001
CAPL-2-KU	9.19	2.01	8.64	2.22	2.23	0.03
PLAYself total	74.51	10.52	64.44	12.64	7.40	0.001
PLAY environment ^{MW}	57.34	16.41	45.86	16.62	5.85	0.001
PLAY self-description ^{MW}	80.30	14.29	64.42	16.50	8.03	0.001
PLAY literacy ^{MW}	79.71	18.64	80.62	19.07	−0.44	0.66
PLAY numeracy ^{MW}	64.94	24.14	64.76	22.79	0.17	0.86
PLAY physical literacy ^{MW}	90.08	13.90	85.22	17.84	2.23	0.03

Legend: ^{MW} indicates variables where differences between groups were calculated by Mann-Whitney test.

Table S2. Descriptive statistics and differences between groups based on age—total sample.

	**Younger Adolescents (*n* = 56)**		**Older Adolescents (*n* = 51)**		***t*-Test/MW Test**	
	**Mean**	**Std.Dev.**	**Mean**	**Std.Dev.**	***t*-Value/Z-Value**	***p***
Body height (cm)	172.53	9.23	173.19	9.59	−0.60	0.55
Body mass (kg)	63.00	14.16	66.32	12.93	−2.12	0.03
Body mass index	21.04	3.62	21.99	3.13	−2.44	0.02
Broad jump (cm)	177.89	29.75	188.04	35.95	−2.65	0.01
Sit and reach (cm)	9.70	8.43	12.51	15.88	−1.89	0.06
Sit-ups (repetitions)	54.17	12.09	58.27	12.99	−2.82	0.01
Beep test (level)	9.48	2.90	9.43	3.07	0.14	0.89
CAPL-2-KU	8.80	2.19	8.98	2.09	−0.73	0.46
PLAYself total	69.13	13.35	69.09	12.15	0.03	0.98
PLAY environment [MW]	50.98	18.15	51.35	16.86	−0.18	0.86
PLAY self-description [MW]	71.92	17.72	71.64	17.16	0.14	0.89
PLAY literacy [MW]	78.54	20.04	81.75	17.58	−1.47	0.14
PLAY numeracy [MW]	65.02	23.50	64.69	23.36	0.12	0.90
PLAY physical literacy [MW]	88.94	16.43	86.09	16.09	1.51	0.13

Legend: [MW] indicates variables where differences between groups were calculated by Mann-Whitney test.

Table S3. Correlations between study variables with participants' age and PE grade.

	**Age**		**PE Grade**	
	**R**	***p***	**R**	***p***
Body height (cm)	0.02	0.76	0.15	0.01
Body mass (kg)	0.18	0.001	−0.04	0.45
Body mass index	0.25	0.001	−0.16	0.01
Broad jump (cm)	0.13	0.02	0.41	0.001
Sit and reach (cm)	0.14	0.01	0.11	0.05
Sit-ups (repetitions)	0.14	0.02	0.47	0.001
Beep test (level)	0.06	0.29	0.42	0.001
CAPL-2-KU	0.17	0.001	0.13	0.02
PLAYself total	−0.01	0.88	0.39	0.001
PLAY environment	0.03	0.60	0.34	0.001
PLAY self-description	−0.06	0.32	0.44	0.001
PLAY literacy	0.06	0.31	−0.07	0.21
PLAY numeracy	0.04	0.52	0.00	0.97
PLAY physical literacy	−0.04	0.51	0.09	0.11

Table S4. Correlations between physical fitness test results and physical literacy.

	**Broad Jump**		**Sit-and-Reach**		**Sit-Ups**		**Beep Test**	
	**R**	***p***	**R**	***p***	**R**	***p***	**R**	***p***
CAPL-2-KU	0.00	0.97	0.11	0.05	0.03	0.61	0.00	0.98
PLAYself total	0.32	0.001	0.08	0.18	0.42	0.001	0.47	0.001
PLAY environment	0.31	0.001	0.07	0.26	0.42	0.001	0.41	0.001
PLAY self-description	0.43	0.001	0.02	0.75	0.51	0.001	0.56	0.001
PLAY literacy	−0.20	0.001	0.12	0.04	−0.08	0.19	−0.10	0.08
PLAY numeracy	−0.01	0.90	0.06	0.29	−0.07	0.26	−0.03	0.64
PLAY physical literacy	−0.01	0.87	0.11	0.07	0.05	0.39	0.17	0.01
